# Synergistic effects of a carbohydrate-controlled diet and *Cuminum cyminum* herbal infusion on metabolic syndrome

**DOI:** 10.3389/fnut.2025.1623478

**Published:** 2025-07-17

**Authors:** Maria Aslam, Habib-ur-Rehman, Tabussam Tufail, Yousaf Almehmadi, Wajd Abdullatif Abualamah, Abdullah R. Alzahrani, Imran Shahid

**Affiliations:** ^1^University Institute of Diet and Nutritional Sciences UIDNS, The University of Lahore UOL, Lahore, Pakistan; ^2^Faculty of Medicine, Rabigh-King Abdulaziz University, Rabigh, Saudi Arabia; ^3^Preventive Medicine Executive Directory Makkah Health Cluster, Makkah, Saudi Arabia; ^4^Department of Pharmacology and Toxicology, Faculty of Medicine, Umm Al-Qura University, Makkah, Saudi Arabia

**Keywords:** carbohydrate diet, *Cuminum cyminum*, metabolic syndrome, BMI, cumin aldehyde, glucose

## Abstract

**Background:**

Metabolic syndrome (MetS) is a growing global health concern and a major risk factor for conditions such as diabetic nephropathy and atherosclerosis. It is marked by chronic inflammation, insulin resistance, and dyslipidemia. Dietary interventions, including carbohydrate-controlled diets, have shown potential in improving metabolic outcomes. *Cuminum cyminum* (cumin), containing the bioactive compound cuminaldehyde, is known for its hypoglycemic and antioxidant properties.

**Aim:**

This study aimed to evaluate the combined effect of a carbohydrate-controlled diet and cumin herbal infusion on metabolic and biochemical parameters in patients with metabolic syndrome.

**Methods:**

A pre- and post-interventional study was conducted on 132 patients (aged 18–60 years) diagnosed with MetS based on the ATP III criteria. Participants were recruited from the University of Lahore Teaching Hospital (ULTH) through purposive sampling and were randomly assigned to control and intervention groups (*n* = 66 each). The intervention group (INEG) received a carbohydrate-controlled diet and cumin herbal infusion for 8 weeks. Anthropometric, biochemical, and physiological parameters were assessed at baseline and post-intervention. A two-way repeated measures ANOVA was used for statistical analysis.

**Results:**

Significant improvements were observed in body mass index (BMI), body weight, and lipid profile parameters (*p* < 0.005). The intervention group showed notable reductions in triglycerides, low-density lipoprotein (LDL), and fasting blood glucose levels compared to the control group (CG).

**Conclusion:**

A carbohydrate-controlled diet combined with cumin herbal infusion may support glycemic control and improve lipid metabolism in individuals with metabolic syndrome. This combined approach shows potential as an adjunct dietary strategy for managing cardiometabolic risk factors.

## 1 Introduction

Metabolic syndrome (MetS) is a multi-component condition characterized by multiple physiological abnormalities, often resulting from a disparity between energy intake and expenditure, and is further influenced by an individual’s genetic and epigenetic factors (1). MetS has emerged as a public health concern in both developed and developing countries. It is characterized by abnormalities such as impaired fasting glucose, central obesity, low high-density lipoprotein (HDL) cholesterol (HDL-C), elevated triglyceride (TG) levels, and hypertension, which cluster together to create a morbid state (2). Metabolic syndrome is linked to a higher likelihood of developing type 2 diabetes mellitus and cardiovascular diseases among 25% of the world’s population. The underlying contributing factors are abdominal adiposity and insulin resistance (3). MetS has been found to have a higher prevalence in men than in women with a heightened likelihood of developing cardiovascular disease, type 2 diabetes, and overall mortality (4). Over the past decade, the gut microbiota has been identified as a major predictor of metabolic conditions such as type 2 diabetes and obesity. Dysbiosis is caused by an obesogenic diet that activates pro-inflammatory processes and causes metabolic endotoxemia, which can promote insulin resistance and cardiometabolic disorders in the population (5). Sedentary lifestyle and excessive calorie intake from a diet high in fats and carbohydrates contribute to vascular alterations and hypercholesterolemia (6). A high-fat diet (HFD) contributes to metabolic disturbances by disrupting redox balance, which weakens the body’s natural antioxidant defense mechanisms against oxidative stress. Additionally, it impairs glucose and lipid regulation and promotes the accumulation of visceral fat (7). Excessive salt consumption has been linked to metabolic diseases, such as insulin resistance, which often coexist with hypertension (8). A high intake of oral sodium in obese individuals is linked to increased urinary sodium excretion, which may contribute to the formation of renal calculi. The kidneys play a key role in regulating electrolyte balance, and excessive sodium intake can lead to hypercalciuria, thereby promoting the formation of calcium stones. In obesity, altered dietary habits and higher metabolic load further disrupt urinary composition. In addition, an elevated body mass index (BMI) itself is an independent risk factor for nephrolithiasis (9). Effective dietary strategies to address metabolic syndrome-associated comorbidities should include lifestyle modifications, such as following a balanced, energy-restricted diet, incorporating functional foods and bioactive nutrients, achieving weight loss, and increasing physical activity (10). Healthy eating and lifestyle changes are the mainstays of metabolic syndrome (MetS) treatment (11). To combat chronic diseases linked to obesity that can jeopardize overall health, strict adherence to healthy eating habits combined with calorie restriction may be beneficial. The Mediterranean diet is considered the best paradigm for evaluating individuals who are overweight or obese (12). Recent studies have shown that cuminaldehyde, a compound found in cumin, exhibits significant *α*-glucosidase and aldose reductase inhibitory activity (13). C*uminum cyminum,* a member of the Apiaceous family, is extensively utilized in Ayurvedic medicine for managing various ailments (14). Cuminaldehyde (4-isopropylbenzaldehyde) is an organic compound classified as a natural aldehyde, with the molecular formula C_10_H_12_O. It is a bioactive component of *Cuminum cyminum, Carum caraway*, and *Cinnamomum verum*, among others. Studies have shown that cuminaldehyde exhibits potent antidiabetic effects in streptozotocin-induced diabetic rats and functions as an effective lipoxygenase inhibitor (13, 15). Zujko et al. (11) investigated the effects of individualized nutrition intervention on reducing the components of metabolic syndrome (MetS) in 90 participants diagnosed with MetS. The participants were assigned to an intervention group (INEG) and a control group (CG) for 3 months. The results showed that individual nutrition education led to improvement in dietary habits, knowledge, and physical activity. The diet modification, which included a higher intake of polyphenols, fiber, PUFA, and PUFA n-3 and a lower intake of SFA, significantly improved the risk factors associated with MetS, such as waist circumference, fasting glucose levels, and HDL cholesterol (11). A 12-month lifestyle program for managing metabolic syndrome (MetS), involving a healthcare team, successfully reversed the participants’ metabolic abnormalities. The study assessed nutrient intake and diet quality over the year, revealing reduced total energy intake, increased consumption of fruits, vegetables, and nuts, and decreased intake of processed and commercially baked foods. Notably, these dietary improvements were observed within the first 3 months and were sustained throughout the 12-month period (16). MetS can be managed through dietary modification. The DASH diet emphasizes the inclusion of fruits, vegetables, and low-fat dairy products while restricting total fat, saturated fat, and sodium intake. This dietary approach helps reduce body weight, lower total and low-density lipoprotein cholesterol (LDL-C), and improve insulin and glucose regulation, as evidenced by previous studies (17). Adherence to the Mediterranean diet has been associated with reductions in body weight and homeostatic model assessment of insulin resistance (HOMA-IR) and improvements in lipid profiles among metabolic syndrome patients, as evidenced by recent studies (18).

Pakistan ranks ninth among the world’s most obese nations, and obesity is associated with a multitude of risk factors, including dyslipidemia. Metabolic syndrome affects 18 to 46% of the Pakistani population. Lifestyle-related risk factors are associated with metabolic syndrome; therefore, addressing these factors is considered a primary focus target for preventive therapies. The researcher aimed to investigate the synergistic effect of a carbohydrate-controlled diet and cumin seed herbal infusion on ameliorating metabolic syndrome, which is a major cause of mortality. Consuming cumin tea regularly, along with diet therapy, leads to improvements in hematological biomarkers and anthropometric measurements.

## 2 Materials and methods

### 2.1 Study design

**Figure 1:**
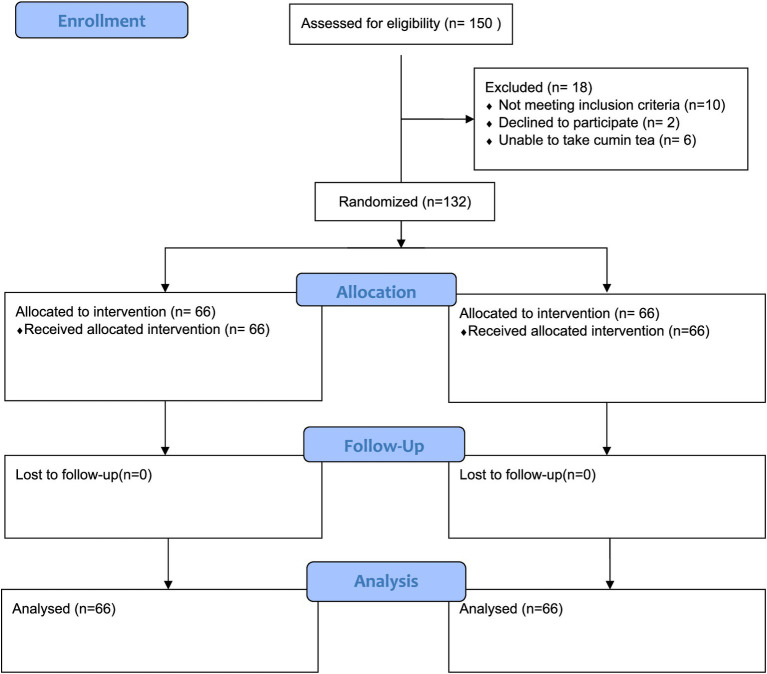
CONSORT flow chart depicting patient allocation.

A pre- and post-interventional trial was carried out to elucidate the synergistic effect of a carbohydrate-controlled diet and *Cuminum cyminum* herbal infusion in metabolic syndrome patients. The study aimed to assess the combined effect of the diet and cumin seed herbal infusion on clinical outcomes in these patients (see Figure 1).

### 2.2 Sampling technique

Patients of either gender, aged 18 to 60 years, and diagnosed with MetS based on the ATP III criteria were recruited through a purposive sampling technique, followed by simple random assignment to control and intervention groups.

### 2.3 Sample selection

#### 2.3.1 Inclusion criteria

Patients of any body mass index with a confirmed diagnosis of MetS who had not used any dietary supplements or adhered to special diets at least 1 month before baseline were included. In addition, patients with at least three symptoms of metabolic syndrome who visited the University of Lahore Teaching Hospital (ULTH) were included and assigned through simple random sampling to control and intervention groups.

#### 2.3.2 Exclusion criteria

Genetically obese individuals, patients already on lipid-lowering drugs according to the recommendation of physicians, those taking any nutritional supplements, individuals allergic to any drugs or specific foods, patients with a medical history of renal diseases, infections, liver disorders, or cardiovascular diseases, patients receiving weight loss drugs or supplements, those undergoing any changes in diet or daily exercise programs, breastfeeding mothers, and pregnant women were excluded from the study.

### 2.4 Sample/cohort size

The sample/cohort size was calculated with a study power of 80% and a significance level of 5%. A total of 132 patients with at least three symptoms of metabolic syndrome who visited the University of Lahore Teaching Hospital (ULTH), Lahore, Pakistan, participated in this research. The Institutional Review Board of the University of Lahore (Ethical code: IRB-UOL-FAHS/808/2021) approved this research. Furthermore, the trial is registered on clinicaltrials.gov (https://clinicaltrials.gov/study/NCT06728449).

### 2.5 Data collection procedure

The selected participants were assigned through simple random sampling to either the control or intervention group. Anthropometric measurements and biochemical tests, such as fasting blood sugar level, lipid profiles (including triglycerides, total cholesterol, LDL, and HDL), liver function tests (LFTs), and renal function tests (RFTs), were taken at the start of the study (0 day) and after 60 days.

### 2.6 Intervention

Following enrollment, the 132 eligible participants diagnosed with metabolic syndrome were randomly assigned to two groups using simple random sampling (*n* = 66 per group). The intervention period lasted for 8 weeks (60 days).

T_0_: Control group receiving a carbohydrate-controlled diet along with conventional therapy.

T_1_: Intervention group receiving the same carbohydrate-controlled diet and conventional therapy, along with cumin seed herbal infusion.

### 2.7 Carbohydrate-controlled diet

Both groups were prescribed individualized meal plans that provided 45–50% of total daily energy from carbohydrates, with emphasis on low-glycemic index foods such as whole grains, legumes, and non-starchy vegetables. Refined sugars, white bread, sweetened beverages, and high-GI foods were limited. Meal distribution and portion sizes were adjusted to maintain glycemic balance throughout the day. Total energy requirements were calculated using the Mifflin-St Jeor equation, and the diet plans were reviewed weekly by a clinical nutritionist to monitor adherence.

### 2.8 Cumin herbal infusion

#### 2.8.1 Formulation

The average weight of one teabag was 2 grams (19). The filter paper was used for packaging the tea bags. *Cuminum cyminum* (cumin seeds) was procured from local markets in Lahore. The cumin seeds were sifted to remove impurities and then coarsely ground for use in the preparation of the tea bags.

The herbal infusion was prepared by placing a cumin tea bag in boiling water and allowing it to steep for 5–7 min before consumption. The patients were instructed to consume the herbal infusion twice daily—once in the mid-morning and once in the evening—using a standard cup size of 200 mL, without adding sugar.

### 2.9 Conventional therapy

All participants continued to receive standard medical care for metabolic syndrome, which included lifestyle counseling and prescribed medications for blood pressure, blood glucose, or cholesterol, as needed. The type and dosage of any medications were not altered by the research team.

### 2.10 Compliance monitoring

Dietary adherence was monitored using weekly food diaries, 24-h dietary recalls, and follow-up visits. Cumin intake compliance was tracked through daily checklists provided to the intervention group. The participants were regularly reminded and motivated through weekly calls or in-person sessions.

### 2.11 Follow-up

During data collection, the patients were informed about weekly follow-up calls using their provided contact numbers. Follow-up calls were conducted every 2 weeks (15 days) to monitor adherence, identify any side effects, and track follow-up or dropout rates. The patients were advised to visit the hospital every 15 days so that compliance could be monitored and cumin seed tea bags could be given for the corresponding days or months. If any issues or side effects were reported, they were treated, adjusted, or eliminated accordingly. After 8 weeks, anthropometric and biochemical tests were conducted to assess the effects of the intervention.

### 2.12 Data analysis

Data were analyzed using SPSS version 24. Two-way repeated measures ANOVA was performed to assess the interaction between time (pre- and post-intervention) and group (control vs. intervention), evaluating differences across the intervention period. The significance level was fixed at a *p-*value of 0.005.

## 3 Results

According to the result, 56% of the patients had three disorders, whereas 44% of the patients had four to five disorders. The mean age of the patients with MetS was 37.54 ± 7.59 years. The age range of the participants was 21–60 years.

### 3.1 Anthropometric measurements

Table 1 shows the changes in the mean scores of BMI and waist circumference in both the control and intervention groups. According to the result, the mean BMI in the control group (T_0_) was 31.44 ± 1.42 before the intervention and 28.48 ± 1.78 after the intervention. In the intervention group (T_1_), the mean BMI was 30.93 ± 1.73 before the intervention and 27.32 ± 1.64 after the intervention. A significant time × group interaction was observed for BMI, with *F* (1, 65) = 5.41 and a *p*-value = 0.023. The mean waist circumference in the control group (T_0_) was 107.83 ± 3.49 before the intervention and 97.87 ± 3.40 after the intervention, while, in the intervention group (T_1_), the mean waist circumference was 108.46 ± 3.77 before the intervention and 97.87 ± 3.40 after the intervention. A significant time × group interaction was observed for waist circumference, with *F* (1, 65) = 9.77 and a *p*-value = 0.003, suggesting that the intervention group had greater improvement compared to the control group.

**Table 1:** Distribution of the participants according to the changes observed in body mass index and waist circumference.

Anthropometric Measurements		Mean ± SD	Percentage change	*F*	df	Significance *p*-value
Body mass index						
T_0_	Pre-intervention	31.44 ± 1.42	9.41	5.41	65	0.023
	Post-intervention	28.48 ± 1.78				
T_1_	Pre-intervention	30.93 ± 1.73	11.67			
	Post-intervention	27.32 ± 1.64				
Waist circumference						
T_0_	Pre-intervention	107.83 ± 3.49	9.24	9.77	65	0.003
	Post-intervention	97.87 ± 3.40				
T_1_	Pre-intervention	108.46 ± 3.77	9.78			
	Post-intervention	97.87 ± 3.40				

### 3.2 Glucose and uric acid levels

Table 2 shows the changes in the mean scores of fasting blood glucose and uric acid levels in both the control and intervention groups. The mean fasting blood glucose level in the control group (T_0_) was 106.27 ± 4.84 before the intervention and 96.39 ± 5.03 after the intervention. In the intervention group (T_1_), the mean fasting blood glucose level was 108.09 ± 4.90 before the intervention and 95.25 ± 4.20 after the intervention. A significant time × group interaction was observed for fasting blood glucose levels, with *F* (1, 65) = 21.10 and a *p*-value < 0.001. The mean uric acid value in the control group (T_0_) was 7.88 ± 0.57 before the intervention and was 7.01 ± 0.47 after the intervention. In the intervention group (T_1_), the mean uric acid value was 8.19 ± 0.55 before the intervention and was 6.99 ± 0.49 after the intervention. A statistically significant interaction was found between time and group for uric acid levels, with *F* (1, 65) = 25.77 and a p-value < 0.001.

**Table 2:** Distribution of the participants according to the changes observed in fasting blood glucose and uric acid levels.

Biochemical tests		Mean ± SD	Percentage change	*F*	df	Significance *p*-value
Fasting blood glucose level						
T_0_	Pre-intervention	106.27 ± 4.84	9.30	21.10	65	0.000
	Post-intervention	96.39 ± 5.03				
T_1_	Pre-intervention	108.09 ± 4.90	11.88			
	Post-intervention	95.25 ± 4.20				
Uric acid level						
T_0_	Pre-intervention	7.88 ± 0.57	11.04	25.77	65	0.000
	Post-intervention	7.01 ± 0.47				
T_1_	Pre-intervention	8.19 ± 0.55	14.65			
	Post-intervention	6.99 ± 0.49				

### 3.3 Lipid profile

Table 3 shows the changes in the mean scores of low- and high-density lipoprotein (LDL/HDL), triglyceride, and total cholesterol levels in both the control and intervention groups. According to the result, the mean LDL level in the control group (T_0_) was 134.09 ± 11.48 before the intervention and 112.43 ± 11.09 after the intervention. In the intervention group (T_1_), the mean LDL level was 131.34 ± 12.89 before the intervention and 107.98 ± 10.76 after the intervention. A significant time × group interaction was observed for LDL levels, with *F* (1, 65) = 11.43 and a *p*-value< 0.001.

**Table 3:** Distribution of the participants according to the changes observed in low- and high-density lipoprotein, cholesterol, and triglyceride levels.

Lipid Profile		Mean ± SD	Percentage change	*F*	df	Significance *p*-value
Low-density lipoprotein level						
T_0_	Pre-intervention	131.34 ± 12.89	16.90	11.43	65	0.000
	Post-intervention	109.15 ± 11.72				
T_1_	Pre-intervention	132.28 ± 12.62	18.54			
	Post-intervention	107.75 ± 11.02				
High-density lipoprotein level						
T_0_	Pre-intervention	48.33 ± 5.71	−20.96	23.50	65	0.000
	Post-intervention	58.46 ± 5.68				
T_1_	Pre-intervention	50.19 ± 4.73	−23.37			
	Post-intervention	61.92 ± 4.75				
Total cholesterol level						
T_0_	Pre-intervention	222.42 ± 10.77	11.08	10.89	65	0.000
	Post-intervention	197.77 ± 11.62				
T_1_	Pre-intervention	222.39 ± 10.33	11.66			
	Post-intervention	196.45 ± 11.67				
Triglyceride level						
T_0_	Pre-intervention	165.44 ± 16.42	7.97	13.61	65	0.000
	Post-intervention	152.24 ± 15.81				
T_1_	Pre-intervention	167.51 ± 15.73	8.80			
	Post-intervention	152.24 ± 15.81				

The mean HDL level in the control group (T_0_) was 48.33 ± 5.71 before the intervention and 58.46 ± 5.68 after the intervention. In the intervention group (T_1_), the HDL level was 50.19 ± 4.73 before the intervention and 61.92 ± 4.75 after the intervention. A significant time × group interaction was observed for HDL levels, with *F* (1, 65) = 23.50 and a *p*-value < 0.001.

The mean triglyceride level in the control group (T_0_) was 167.51 ± 15.73 before the intervention and 152.77 ± 15.07 after the intervention. In the intervention group (T_1_), the mean triglyceride level was 165.43 ± 16.42 before the intervention and 152.24 ± 15.81 after the intervention. A significant time × group interaction was observed for triglyceride levels, with *F* (1, 65) = 13.61 and a *p*-value < 0.001.

The mean cholesterol level in the control group (T_0_) was 222.42 ± 10.77 before the intervention and 197.77 ± 11.62 after the intervention, with T_0_*: t* (65) = 55.34 and a p-value< 0.001. In the intervention group (T_1_), the mean cholesterol level was 222.39 ± 10.33 before the intervention and 196.45 ± 11.67 after the intervention, with *F* (1, 65) = 10.89 and a *p*-value < 0.001.

## 4 Blood pressure

Table 4 shows the changes in the mean scores of blood pressure readings in both the control and intervention groups. In the control group, the mean systolic blood pressure was 146.34 ± 5.25 before the intervention and 142.00 ± 4.88 after the intervention. In the intervention group (T_1_), the mean systolic blood pressure was 144.18 ± 3.29 before the intervention and 136.84 ± 3.98 after the intervention. A statistically significant interaction was found between time and group for systolic blood pressure, with *F* (1, 65) = 86.28 and a *p*-value < 0.001.

**Table 4:** Distribution of the participants according to the changes observed in blood pressure readings.

Blood Pressure Readings		Mean ± SD	Percentage CHANGE	*F*	df	Significance *p*-value
Systolic blood pressure reading						
T_0_	Pre-intervention	146.34 ± 5.25	2.96	86.28	65	0.000
	Post-intervention	142.00 ± 4.88				
T_1_	Pre-intervention	144.18 ± 3.29	5.09			
	Post-intervention	136.84 ± 3.98				
Diastolic blood pressure reading						
T_0_	Control group pre-intervention	87.45 ± 2.13	4.44	98.82	65	0.000
	Control group post-intervention	83.56 ± 2.63				
T_1_	Cumin group pre-intervention	87.41 ± 2.17	10.01			
	Cumin group post-intervention	78.66 ± 2.95				

In the control group, the mean diastolic blood pressure was 87.45 ± 2.13 before the intervention and 83.56 ± 2.63 after the intervention. In the intervention group (T_1_), the mean diastolic blood pressure was 87.41 ± 2.17 before the intervention and 78.66 ± 2.99 after the intervention. A statistically significant interaction was found between time and group for diastolic blood pressure, with *F* (1, 65) = 98.82 and a *p*-value < 0.001, indicating a substantial difference in the pre- to post-intervention changes between the intervention and control groups.

### 4.1 Liver and renal function tests

Table 5 shows the changes in the mean scores of liver enzyme levels in both the control and intervention groups. In the alkaline phosphatase (ALP) test, the mean level in the control group (T_0_) was 128.30 ± 4.75 before the intervention and 120.13 ± 4.22 after the intervention. In the intervention group (T_1_), the mean level was 128.30 ± 4.75 before the intervention and 118.03 ± 3.49 after the intervention. A statistically significant interaction was found between time and group for ALP levels, with *F* (1, 65) = 14.45 and a *p*-value < 0.001. In the aspartate aminotransferase (AST) test, the pre-intervention mean score in the control group(T_0_) was 58.13 ± 6.08 and the post-intervention mean score was 50.19 ± 4.73. In the intervention group (T_1_), the mean level was 58.54 ± 6.21 before the intervention and 49.53 ± 4.96 after the intervention. There was no significant interaction between time and group for AST levels, with *F* (1, 65) = 7.25 and a *p*-value > 0.005.

**Table 5:** Distribution of the participants according to the changes observed in liver enzyme levels and renal functioning.

Hepatorenal Parameters		Mean ± SD	Percentage change	*F*	df	Significance *p*-value
LFT						
Alkaline Phosphatase (ALP) Test						
T_0_	Pre-intervention	128.30 ± 4.75	6.36	14.45	65	0.000
	Post-intervention	120.13 ± 4.22				
T_1_	Pre-intervention	128.30 ± 4.75	8.00			
	Post-intervention	118.03 ± 3.49				
Aspartate Aminotransferase (AST) Test						
T_0_	Pre-intervention	58.13 ± 6.08	13.66	7.25	65	0.009
	Post-intervention	50.19 ± 4.73				
T_1_	Pre-intervention	58.54 ± 6.21	15.39			
	Post-intervention	49.53 ± 4.96				
RFT						
Change in Blood Urea Nitrogen (BUN) Level						
T_0_	Pre-intervention	50.25 ± 4.89	16.58	96.20	65	0.000
	Post-intervention	41.92 ± 4.75				
T_1_	Pre-intervention	50.03 ± 5.23	31.48			
	Post-intervention	34.28 ± 4.42				
Serum creatinine level						
T_0_	Pre-intervention	1.53 ± 0.23	7.19	5.32	65	0.024
	Post-intervention	1.42 ± 0.23				
T_1_	Pre-intervention	1.54 ± 0.22	12.33			
	Post-intervention	1.35 ± 0.18				

In the control group (T_0_), the mean blood urea nitrogen (BUN) level was 50.25 ± 4.89 before the intervention and 41.92 ± 4.75 after the intervention. In the intervention group (T_1_), the mean BUN level was 50.03 ± 5.23 before the intervention and 34.28 ± 4.42 after the intervention. A significant time × group interaction was observed for BUN levels, with *F* (1, 65) = 96.20 and a *p*-value < 0.001, suggesting that the intervention group had greater improvement compared to the control group.

According to the result, the mean serum creatinine level in the control group (T_0_) was 1.53 ± 0.23 before the intervention and 1.42 ± 0.23 after the intervention. In the intervention group (T_1_), the mean serum creatinine level was 1.54 ± 0.22 before the intervention and 1.35 ± 0.18 after the intervention. No significant time × group interaction was observed for serum creatinine levels, with *F* (1, 65) = 5.32 and a *p*-value > 0.005, suggesting that the intervention group had greater improvement compared to the control group.

## 5 Discussion

The current study aimed to determine the combined effect of diet and cumin herbal tea on the clinical outcomes of metabolic syndrome, including abdominal obesity, dyslipidemia, hypertension, and insulin resistance. The findings demonstrated that integrating cumin tea with a carbohydrate-controlled dietary approach significantly enhanced metabolic outcomes compared to either intervention alone. Metabolic syndrome is a chronic disease with lifestyle interventions serving as a foundation of treatment. Nutritional interventions, focused primarily on weight loss, are ineffective in addressing all risk factors. Diet quality is also a major contributor to reducing the risk of metabolic syndrome (20).

The participants in the combined intervention group exhibited substantial improvements in fasting blood glucose levels, as evidenced by the results (*F* (1, 65) = 21.10, *p* < 0.001). The results are based on the hypoglycemic effects of cumin, attributed to its bioactive compound cuminaldehyde, which improves glucose metabolism and reduces oxidative stress. The carbohydrate-controlled diet complements this by reducing postprandial glucose levels and promoting a stable insulin response through the inclusion of low glycemic index foods. The combined intervention also led to significant reductions in triglycerides and increases in HDL cholesterol. These lipid improvements likely resulted from the synergistic effects of cumin’s lipid-lowering properties and the metabolic benefits of carbohydrate restriction. A study reported by Zare et al. found that the administration of cumin (3gm /day) to overweight and obese women resulted in reductions in serum fasting cholesterol levels, triglycerides, weight, body mass index, waist circumference, and body fat percentage. It also enhanced the concentration of high-density lipoprotein and improved anthropometric parameters (21).

Taghizadeh et al. (22) conducted a study on 72 overweight participants to investigate the combined impact of *Cuminum cyminum* L. and lime, administered in doses of 75 mg and 25 mg, respectively, over 8 weeks. The higher dose of the nutraceutical (cumin and lime) resulted in declines in fasting plasma glucose levels (*p* < 0.001), serum triglycerides (*p* = 0.03), total cholesterol levels (*p* = 0.004), and LDL cholesterol levels (*p* = 0.01) (22).

Aktas et al. conducted a study on 80 obese participants to investigate the impact of a low-calorie diet and physical activity on body weight over 12 weeks. Turkish Muslims residing in Germany are prone to developing obesity, type 2 diabetes mellitus, and metabolic syndrome. The results revealed that tailored lifestyle interventions led to a significant reduction of 6% in body weight and improvements in the levels of glucose, glycosylated hemoglobin, and cholesterol (23).

Abdominal obesity, measured by waist circumference and BMI, improved significantly in the combined intervention group compared to the control group. While carbohydrate restriction promoted fat loss through reduced calorie intake and enhanced satiety, cumin’s thermogenic properties and ability to regulate fat metabolism amplified these results (*p* < 0.001). Karpagam and Priya (24) conducted a human trial to evaluate the impact of *Cuminum cyminum* combined with lime water on obesity. Participants consumed the preparation every morning on an empty stomach for 3 weeks. The preparation involved boiling 200 mL of water with 2 grams of cumin seeds, allowing it to steep overnight, and then adding the juice of half a lime before consumption. The findings demonstrated that the *Cuminum cyminum* and lime water combination effectively reduced overweight and obesity (*p* < 0.005) (24). Cumin supplementation has also been linked to a decrease in body mass index (BMI) (25).

Cumin (*Cuminum cyminum L*.) supplementation significantly reduces total cholesterol and low-density lipoprotein cholesterol while increasing high-density lipoprotein cholesterol. It shows no significant effect on triglyceride levels, except in individuals without hypertriglyceridemia, indicating its efficacy in managing lipid profiles. Cumin supplementation resulted in a significant reduction in TC, with studies reporting mean differences of −3.96 mg/dL and −10.90 mg/dL. Cumin was effective in lowering LDL-C, with reductions of −6.94 mg/dL noted. An increase in HDL-C levels was observed, with a mean difference of 3.35 mg/dL (26). Cumin supplementation has been associated with a significant decrease in total cholesterol levels, with studies reporting reductions ranging from approximately 3.96 mg/dL to 10.90 mg/dL (25, 26).

*Cuminum cyminum* supplementation has been shown to lower total cholesterol and BMI. It also improves waist circumference, high-density lipoprotein, and triglyceride levels, although its effect on low-density lipoprotein is inconclusive without longer supplementation periods. Cumin supplementation resulted in a significant reduction in TC, with studies reporting a mean difference of −3.96 mg/dL (25).

Notable improvements in blood pressure were observed, with greater reductions in systolic and diastolic blood pressure observed in the combined intervention group. The antihypertensive effects of cumin, attributed to its antioxidant properties and enhancement of nitric oxide-mediated vasodilation, likely complimented the weight loss and metabolic improvements induced by the carbohydrate-controlled diet. Together, these interventions may improve endothelial function and reduce arterial stiffness over time. Although cumin exhibits promising nephroprotective properties, a study conducted by Alfahdawi et al. (27) investigated the effect of an aqueous extract of cumin seeds on kidney function in albino rats. The results showed improvements in kidney function, with a *p*-value of <0.05 (27). Similar results were reported by Kumar et al. (28), where the administration of *Cuminum cyminum* aqueous extract significantly restored elevated levels of urea, uric acid, and creatinine in mice (28).

In conjunction with exercise, cumin extract has been shown to improve insulin resistance, further supporting metabolic health. Cumin extract consumption, when combined with pilates training, significantly improved lipid profiles in overweight and obese women. The intervention led to decreased serum triglyceride, cholesterol, and LDL levels, while increasing HDL levels, as observed in a previous study (29).

*Cuminum cyminum* exhibits a hepatoprotective effect, as evidenced by significant reductions in liver enzyme levels. This is demonstrated by the results for ALP: *F* (1, 65) = 14.45, *p* < 0.001. *Cuminum cyminum* seeds powder exhibits hepatoprotective properties, suggesting it may aid in liver function recovery following acetaminophen-induced injury. This indicates potential therapeutic benefits for liver health, particularly in mitigating damage caused by certain pharmaceutical agents (30). Previous research indicates that cumin seed powder supplementation can prevent non-alcoholic fatty liver disease and reduce oxidative stress in rats that are fed a high-fat diet, suggesting a potential protective effect on liver function related to dietary fat intake (31).

This study’s key strength lies in the novel exploration of the combined effects of two complementary interventions, providing a comprehensive approach to managing metabolic syndrome-associated comorbidities. The findings of the study underscore the potential of integrating functional foods such as cumin into tailored dietary strategies for metabolic syndrome management. This discussion highlights the potential synergistic effects of cumin and a carbohydrate-controlled diet, emphasizing their complementary roles in improving metabolic health.

## 6 Conclusion

The findings of this study suggest that combining a carbohydrate-controlled diet with *Cuminum cyminum* (cumin) infusion may support improvements in metabolic parameters among individuals with metabolic syndrome. Notable reductions were observed in lipid profile markers, including total cholesterol, LDL, and triglycerides, along with modest improvements in renal function indicators. These outcomes may be attributed in part to the bioactive compounds in cumin, such as cuminaldehyde, which are known for their antioxidant and anti-inflammatory properties. While the results are promising, the intervention should be considered a complementary approach alongside standard dietary and lifestyle modifications. Additional long-term, blinded, and standardized studies are needed to confirm these findings and clarify the role of cumin as an adjunct therapy in managing metabolic syndrome.

### 6.1 Limitations

This study has several limitations. The purposive sampling method may limit the generalizability of the results. The open-label design without blinding might have introduced bias. Cumin infusion was not standardized for active compounds, restricting the ability to attribute observed effects to specific phytochemicals. The short intervention period (8 weeks) limits insights into long-term outcomes. Compliance was based on self-reported dietary records, which might not have been fully accurate. Additionally, variations in participants’ medications were not controlled and might have influenced the findings.

## Data Availability

The original contributions presented in the study are included in the article/supplementary material, further inquiries can be directed to the corresponding author.
